# Multidermatomal Shingles Occurrence Post-therapeutic Dry Needling (TDN) in a Saudi Woman

**DOI:** 10.7759/cureus.25309

**Published:** 2022-05-24

**Authors:** Tara Z Alfaddagh, Sahar Al-Natour

**Affiliations:** 1 Medical School, Imam Abdulrahman Bin Faisal University, Dammam, SAU; 2 Dermatology, College of Medicine, Imam Abdulrahman Bin Faisal University, Dammam, SAU

**Keywords:** varicella, zoster, shingles, therapeutic, dry needling

## Abstract

This is a case report of the occurrence of shingles (herpes zoster) in a multidermatomal distribution following therapeutic dry needling. The patient developed acute reactivation of varicella-zoster virus two days after the third session of dry needling for the relief of long-standing pain in the hip and sacroiliac joints. The lesions initially presented with severe pain along lumbar dermatomes with later rash development. The patient was treated with oral valacyclovir 1,000 mg three times daily for seven days, and the pain was controlled with ibuprofen. The lesions resolved two weeks later without any complications or sequelae.

## Introduction

Therapeutic dry needling (TDN) is a relatively new intervention performed by physiotherapists to help relieve musculoskeletal pain, particularly when other pain-relieving interventions have failed. The procedure is performed using a thin solid needle that penetrates the skin and stimulates the underlying myofascial trigger points and muscular and connective tissues to elicit a neuromuscular pain-reducing response and the resulting movement impairment [1]. The concept of needling myofascial trigger points to reduce pain dates to the early 1940s and was pioneered by Dr. Janet Travell [2]. Her method is referred to as “wet needling” which utilizes a hollow hypodermic needle to inject pain relievers, corticosteroids, or Botulinum toxin into neuromuscular tissue. The two methods are distinctly different primarily in the type of needle used in the intervention and the intention of the insertion of the needle [2]. Dry needling elicits a pain-reducing response by stimulating the neuromuscular tissue versus a direct anti-inflammatory or muscular response mediated by injection of a drug into the trigger point in wet needling.

There is a paucity of literature on the adverse events associated with TDN. Furthermore, much of the published literature concerning the adverse events of TDN is extrapolated from acupuncture literature, which is distinctly different, in the proposed mechanisms and applications. Although acupuncture is widely used in the management and relief of pain associated with shingles, paradoxically one case has recently been described as being associated with herpes zoster (HZ) reactivation [3].

To our knowledge, no reports of reactivation of HZ after dry needling have been described to date. Hence, we present a case of shingles following TDN.

## Case presentation

A 53-year-old female with a history of severe bilateral hip and sacroiliac joint pain for 10 months, not responsive to steroid injections, non-steroidal anti-inflammatory drugs (NSAIDs), and physiotherapy, underwent three dry needling sessions within a nine-day period. The patient had a history of varicella-zoster virus (VZV) infection as a child and no prior shingles vaccinations. She was not known to have immunodeficiency nor was she on any immunosuppressants.

The needle was inserted into the piriformis, gluteus medius, and minimus muscles. During the first session, needle manipulation therapy was performed bilaterally. For the following two sessions, only the left side was treated. Minimal pain, bleeding, and slight bruising were experienced at the time of treatment, all of which quickly subsided. The treatment succeeded in temporarily reducing the hip and sacroiliac pain, for which it was intended.

Two days after the third session of TDN, a sharp burning pain developed along the anterior aspect of the left thigh radiating from the groin area. The pain gradually worsened over the following two days with a 5/10 pain severity grade. The pain was worse at night. Although there was no itching, redness, or rash at the site of pain initially, two days later, she noticed three small red papules on the left inner thigh. The following day, the eruption spread to involve the left mid-thigh and upper buttock. Before presenting to a dermatologist, she self-treated with fusidic acid and betamethasone cream for possible folliculitis. The lesions started blistering and growing in number and size over the next day or two.

**Figure 1:**
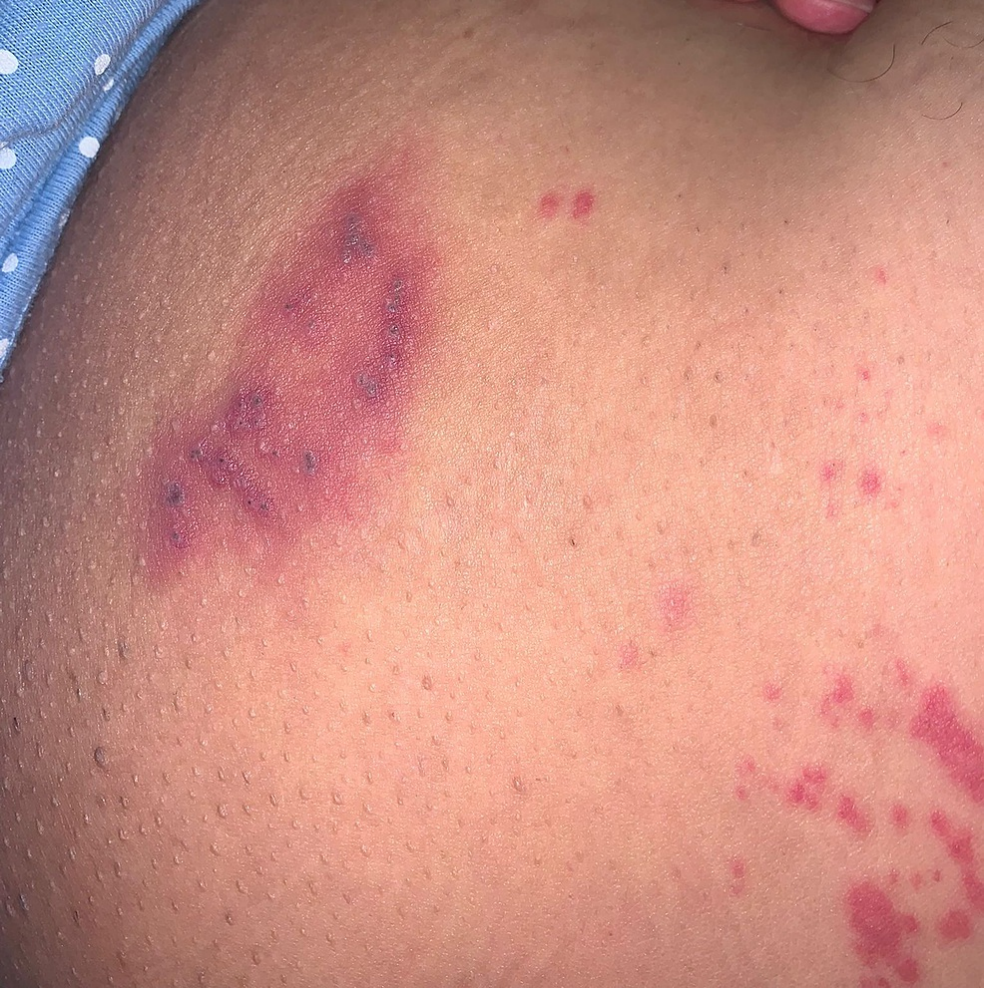
Clustered erythematous papules and vesicles and bruising on the left inner thigh on the L2 dermatome.

**Figure 2:**
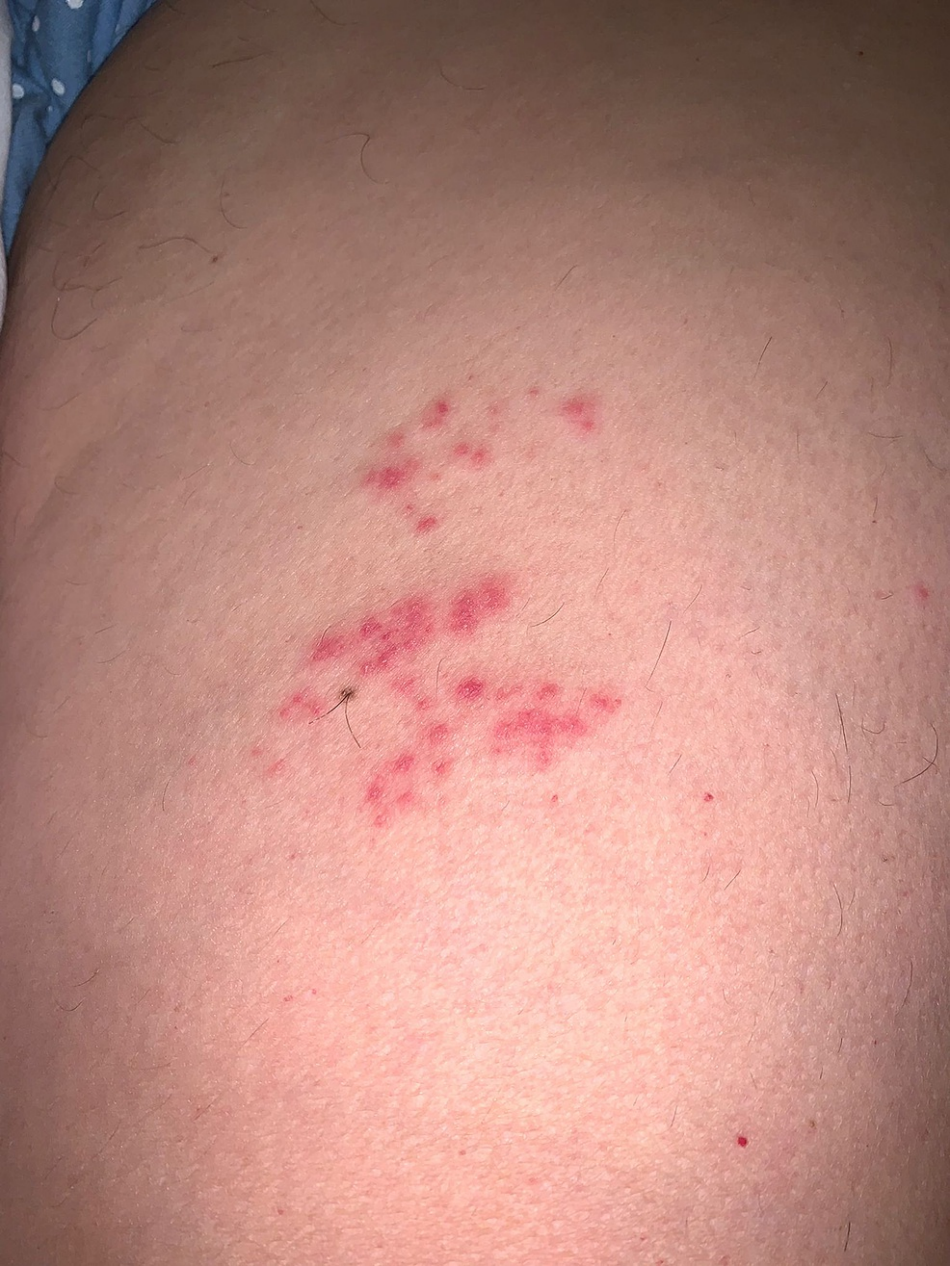
Grouped papules and vesicles on the left anterior mid-thigh on the L3 dermatome.

**Figure 3:**
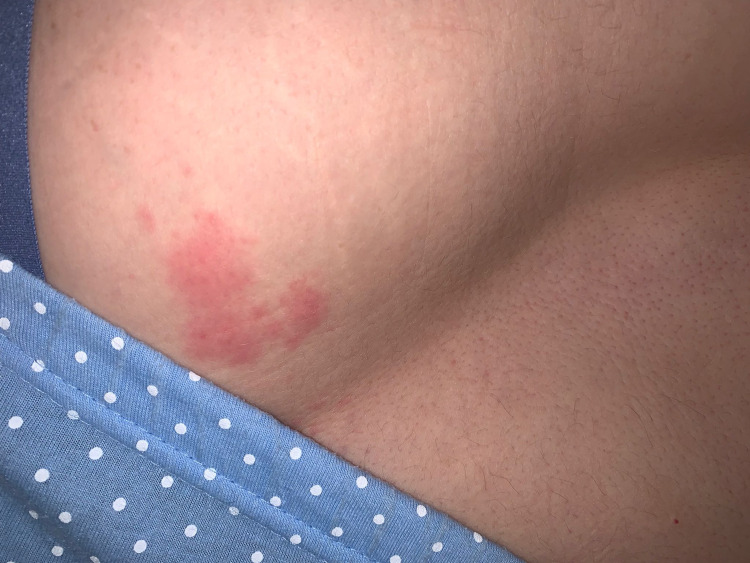
Early lesions over the left upper buttock and characteristically not crossing the midline on the L1 dermatome.

On examination, clustered erythematous vesicular eruptions were seen on the left thigh medially on L2 dermatome, anterior mid-thigh on L3 dermatome, and posteriorly over the upper gluteal region on L1 dermatome (Figures 1-3, respectively). Tender inguinal lymphadenopathy was also present. The clinical morphology of the lesions was consistent with HZ, and the polymerase chain reaction result was positive for VZV, indicating a diagnosis of shingles.

On day four of the eruption, she was started on valacyclovir 1,000 mg, three times a day, for seven days, and ibuprofen 600 mg three times a day for 10 days for analgesia. By day four of treatment, the pain started to decrease but new eruptions continued. Within two weeks of starting antiviral therapy, the pain and rash had subsided with no sequelae. She continues to be symptom-free five months later.

## Discussion

During the time of researching materials for this study, the authors found a scarcity of published literature about the adverse effects of TDN, a relatively new therapy used to improve pain and movement. Two published studies have documented the adverse events associated with TDN [4,5]. Both studies listed the most common minor adverse events as bleeding, bruising, and pain during dry needling sessions, paralleling the same adverse events in our case. The study published by Brady et al. in 2014 also listed some less common effects such as aggravation of symptoms, headache, nausea, and numbness [4]. While the more recent study published in 2020 by Boyce et al. listed a few other rare major adverse effects of pneumothorax and excessive bleeding [5]. Fortunately, none of these more serious adverse effects were witnessed in our patient.

At the time of this study, a literature search by the authors did not result in any articles documenting the occurrence of HZ or its association with TDN. However, several reports have documented HZ activation post-muscular and nerve manipulation [3,6,7]. One case study reported a case of HZ post-acupuncture performed for relieving back pain [3]. Another published case study showed accidental widespread autoinoculation of varicella following the use of a home microneedling device [6]. A third study documented a case of HZ infection precipitated by surgical manipulation of the trigeminal nerve during an attempted microvascular decompression procedure [7].

Shingles occur when the previously dormant VZV in a ganglion or nucleus is reactivated and then travels to the corresponding dermatome, resulting in pain and rash. Triggers for shingles include stress, trauma, disease, and immune disorders [3]. The 53-year-old woman in this study was healthy, without any known diseases or immunodeficiency disorders, and did not report any extraordinary stress factors that may have contributed to the onset of shingles.

While therapeutic dry needling, acupuncture, and home microneedling are completely different procedures, they all represent physical trauma, which along with surgical procedures are known to precipitate HZ eruptions [8].

## Conclusions

This case study documents the occurrence of HZ following TDN in an individual with no known risk factors. Research on adverse events related to dry needling is continuously evolving. This case could provide a basis for future reference, in addition to providing advice for early detection, recognition, and treatment of HZ occurring directly after TDN.
